# Feasibility between Bifidobacteria Targeting and Changes in the Acoustic Environment of tumor Tissue for Synergistic HIFU

**DOI:** 10.1038/s41598-020-64661-6

**Published:** 2020-05-08

**Authors:** Die Xu, Wenjuan Zou, Yong Luo, Xuan Gao, Binglei Jiang, Yaotai Wang, Fujie Jiang, Jie Xiong, Chun Chen, Yu Tang, Hai Qiao, Huanan Li, Jianzhong Zou

**Affiliations:** 1 State Key Laboratory of Ultrasound Engineering in Medicine Co-Founded by Chongqing and the Ministry of Science and Technology, Chongqing Collaborative Innovation Center for Minimally-invasive and Noninvasive Medicine, College of Biomedical Engineering, Chongqing Medical University, Chongqing, 400016 China; 2 Chongqing Traditional Chinese Medicine Hospital, Chongqing, 400021 China

**Keywords:** Biomedical engineering, Biomedical engineering, Biomedical engineering, Biomedical engineering, Biomedical engineering

## Abstract

High intensity focused ultrasound (HIFU) has been recently shown as a rapidly developing new technique for non-invasive ablation of local tumors whose therapeutic efficiency can be significantly improved by changing the tissue acoustic environment (AET). Currently, the method of changing AET is mainly to introduce a medium with high acoustic impedance, but there are some disadvantages such as low retention of the introduced medium in the target area and a short residence time during the process. In our strategy, anaerobic bacterium Bifidobacterium longum (*B. longum)* which can colonize selectively in hypoxic regions of the animal body was successfully localized and shown to proliferate in the hypoxic zone of tumor tissue, overcoming the above disadvantages. This study aimed to explore the effects of Bifidobacteria on AET (including the structure and acoustic properties of tumor tissues) and HIFU ablation at different time. The results show that the injection of Bifidobacteria increased the collagen fibre number, elastic modulus and sound velocity and decreased neovascularization in tumor tissues. The number of collagen fibres and neovascularization decreased significantly over time. Under the same HIFU irradiation intensity, the *B. longum* injection increased the coagulative necrosis volume and decreased the energy efficiency factor (EEF). This study confirmed that Bifidobacteria can change the AET and increase the deposition of ultrasonic energy and thereby the efficiency of HIFU. In addition, the time that Bifidobacteria stay in the tumor area after injection is an important factor. This research provides a novel approach for synergistic biologically targeted HIFU therapy.

## Introduction

Malignant tumors are diseases that seriously threaten human health. Although traditional methods of tumor therapy have been widely used and proved to be effective in treating cancerous tumors, there are inevitable side effects, including immune system destruction, high costs and low treatment efficiency^1,2^. High-intensity focused ultrasound (HIFU) is considered a new technique for the non-invasive ablation of solid tumors^3,4^, which has recently attracted tremendous attention. However, there are series of challenges in the treatment process. For example, when it comes to deep-seated tumor tissue, the ultrasonic energy decreases exponentially with increasing penetration depth. In addition, heterogeneous blood perfusion rates can eliminate some of the temperature elevation and reduce the energy deposition in the target area, thereby reducing the therapeutic efficiency of HIFU significantly^5,6^. By increasing the output acoustic power or prolonging the sonication time, these limitations can be overcome, but these options come with an increased the risk of adverse biological effects^7–9^.

Changing the tissue acoustic environment (AET) is one approach to maximize the benefits of HIFU treatment and minimize the side effects. The AET refers to the structure, density and/or acoustic properties of the tumor tissue that is altered during HIFU treatment^10^. The currently available methods for changing the AET include increasing the acoustic impedance reflection interface^11,12^, reducing blood perfusion^13,14^, and enhancing the cavitation effects^15,16^. However, each of these methods has certain disadvantages, such as a complicated operation and poor stability and controllability^17^. Therefore, this study is intended to find a new, safe, controllable and easy-to-use way to change the AET in order to increase the therapeutic efficiency of HIFU.

Bifidobacteria are probiotics with facultative anaerobic properties. After entering the bloodstream, Bifidobacteria penetrate into tissue through the fragile areas of the blood vessels or remain in the tumor blood vessels separate from the normal proximal blood vessels^18–20^. Bifidobacteria easily colonize and propagate in the necrotic areas of tumor tissue, inhibit blood vessel growth in tumors and promote tumor cell necrosis^20–25^. Bifidobacteria are currently being used in gene-related and drug loading studies^23,26–31^. Our research group has used Bifidobacterium as a targeting carrier to deliver HIFU synergistic materials^32,33^). However, Bifidobacterium has not as a biological targeting material that is directly used to change the AET of tumors for synergistic HIFU, and no related studies have been performed at home or abroad.

In this study, *Bifidobacterium longum (B. longum)* was selected as a targeting biomaterial for injection into tumor-bearing mice. The effects of Bifidobacteria on the AET and the efficiency of ultrasonic energy conversion and deposition by HIFU ablation were detected at different time points. The AET mainly includes two aspects, acoustic properties and tissue microenvironment. In our study, we mainly explored the sound velocity, sound attenuation, neovascularization, collagen fibres and elastic modulus (which closely related to collagen fibres). This study lays a foundation for the application of Bifidobacteria as a long-lasting HIFU synergist.

## Results

### Targeting

**Figure 1:**
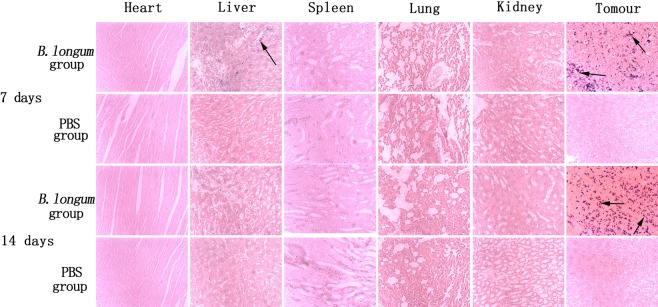
Gram staining of pathological tissue for evaluating the growth and distribution of *Bifidobacterium longum* in various normal tissues (heart, liver, spleen, lung, and kidney) and tumors (200× magnification). The Bifidobacteria are observed by blue staining, as indicated by the arrows.

Seven days after the first injection, a few blue-violet-stained Bifidobacteria were scattered in the livers of 2 mice and tumors of 20 mice in the *B. longum* group. Fourteen days after the first injection, no Bifidobacteria were observed in the livers of mice, and Bifidobacteria were found in all the tumors. Blue-violet-stained Bifidobacteria were not found in the PBS group. The dyeing results are shown in Fig. 1.

### AET

#### Sound velocity

**Figure 2:**
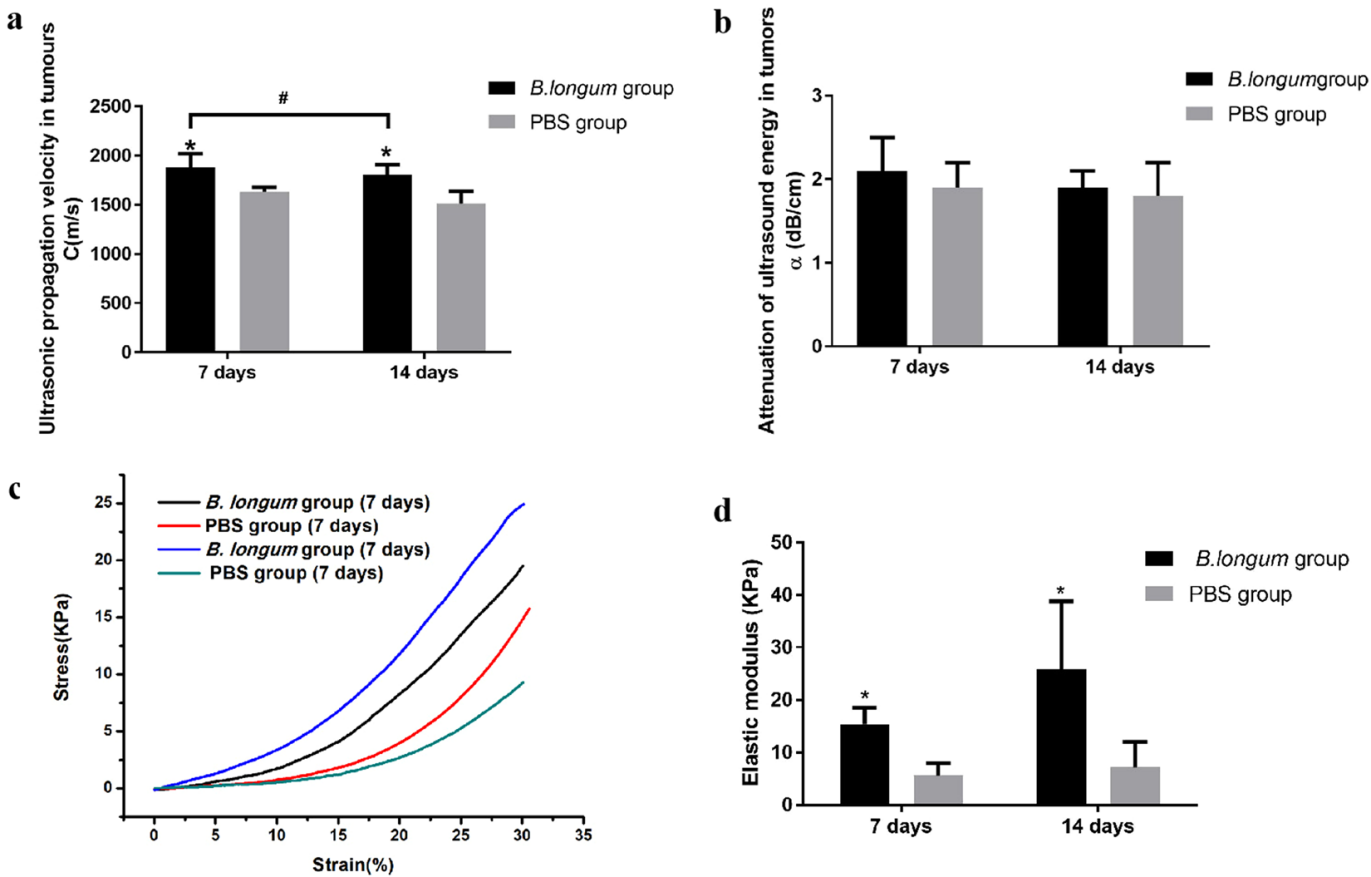
(**a**) The comparison of ultrasound velocity in tumor tissues in each group. **(b)** The comparison of attenuation of ultrasound energy in tumor tissues in each group.Attenuation of ultrasound energy in tumor tissue (n=10). **(c)** Stress-strain curve obtained by biomechanical testing (n=10). **(d)** The comparison of elastic modulus in tumor tissues in each group.

The sound velocities in tumors isolated from mice belonging to the *B. longum* and PBS groups 7 days after the first injection were 1880 ± 140 m/s and 1630 ± 50 m/s, respectively. The sound velocity in tumors was higher in the *B. longum* group than in the PBS group (*P* = 0.003). The sound velocities in tumors isolated from mice belonging to the *B. longum* and PBS groups 14 days after the first injection were 1810 ± 100 m/s and 1510 ± 120 m/s, respectively, and those in tumors was higher in the *B.longum* group than in the PBS group (*P* = 0.043). There were significant differences in sound velocity between samples obtained in the two *B. longum* group at 1 and 2 weeks (*P* = 0.042; Fig. 2a).

#### Sound attenuation

Acoustic attenuation values of (2.1 ± 0.4) dB/cm and (1.9 ± 0.3) dB/cm were found for tumors in the *B. longum* and PBS groups 7 days after the first injection, respectively, and values of (1.90 ± 0.20) dB/cm and (1.8 ± 0.4) dB/cm were obtained fourteen days after the first injection, respectively. No significant differences in acoustic attenuation were found between the two groups (*P* > 0.05), and no significant differences in tumor attenuation were between samples obtained at 1 and 2 weeks for either group (*P* > 0.05; Fig. 2b).

#### Tissue hardness–Elastic modulus

The stress-strain relationship determined by testing the compression of the isolated tumor tissues is shown in Fig. 2c. The first 10% of the curve is approximately linear, and thus, the first 10% of each sample was used for linear fitting to obtain the elastic modulus. The results are shown in Fig. 2d. The elastic modulus obtained for the *B. longum* group 1 and 2 weeks after the injection was (15 ± 3) kPa and (26 ± 13) kPa, respectively, and these values were significantly greater than those found for the PBS group at the same time (*P* = 0.000; *P* = 0.003). The elastic modulus calculated for the *B. longum* group 1 and 2 weeks after the injection showed no significant differences between each other (*P*å 0.05).

#### Pathology

**Figure 3:**
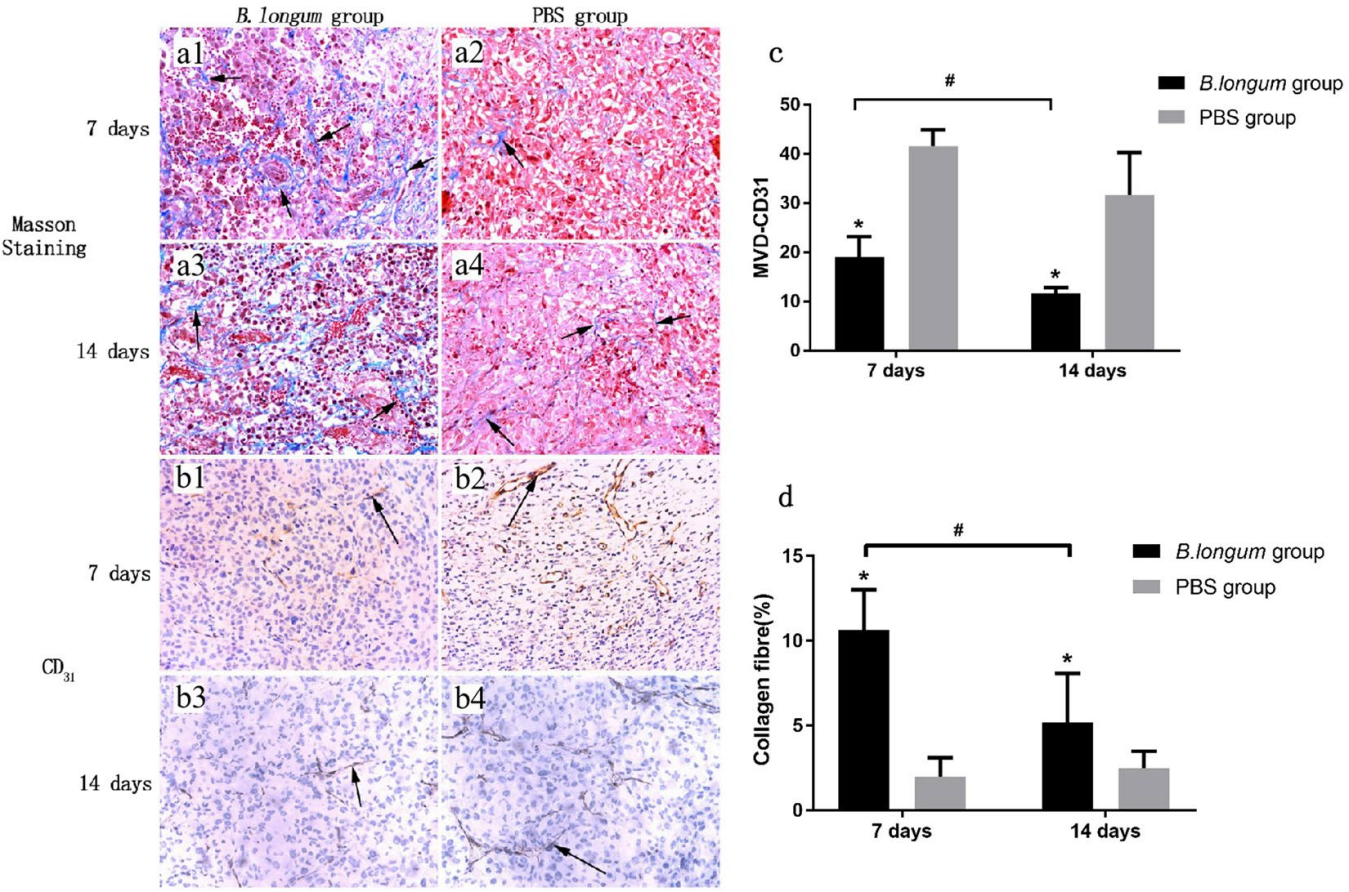
(**a1-a4**) Masson staining of tumor tissues (200× magnification). The blue areas indicated by the arrows are collagen fibres. (**b1-b4**) Immunohistochemistry examination of tumor tissues (CD31 staining; 200× magnification). The arrows indicate new blood vessels. (**c**) The comparison of collagen fibre content of tumor tissues in each group. (**d**) The comparison of neovascular density of tumor tissues in each group.

A large number of collagen fibres were densely arranged in the tissue of the *B. longum* group (Fig. 3a1,a3). The collagen fibre content percentages in the tissue 1 and 2 weeks after the injection were 11 ± 2 and 5 ± 3, respectively, and these values are significantly different (*P* = 0.000; Fig. 3c). In the PBS group, a small number of collagen fibres were detected, and these showed a scattered distribution (Fig. 3a2,a4). Further analysis revealed that the PBS group exhibited a significantly fewer amount of collagen fibres than the *B. longum* group at the same time points (*P* = 0.000; *P* = 0.018; Fig. 3c). One and 2 weeks after the injection, the *B. longum* group exhibited sparse blood vessels and incomplete vascular structures (Fig. 3b1;b3). Specifically, the numbers of new blood vessels in this group were 19 ± 4 and 12 ± 1, and these values are significantly different (*P* = 0.021; Fig. 3d). In the PBS group, the vascular structures were nearly complete 1 and 2 weeks after the injection and showed a long and round shape (Fig. 3b2;b4). In addition, the PBS groups showed a significantly higher number of new blood vessels than the *B. longum* group at the same time points (*P* = 0.034; *P* = 0.04; Fig. 3d).

#### Synergistic HIFU therapy

**Figure 4:**
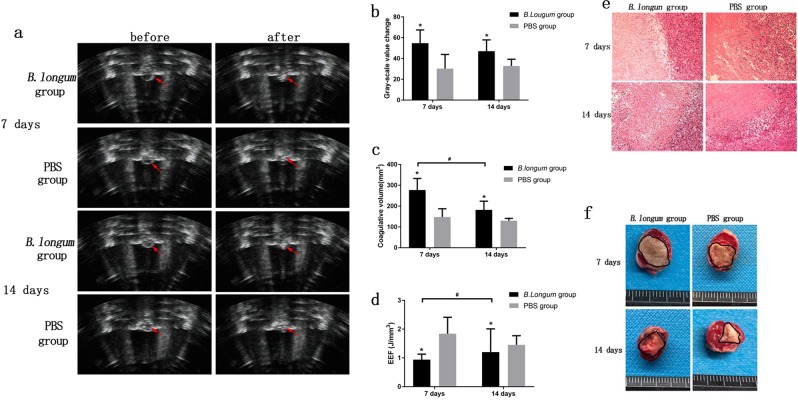
(**a**) *In vivo* ultrasound imaging of tumors (indicated by red arrows) before and after HIFU ablation. **(b)**The comparison of gray values of tumor tissues in each group after HIFU irradiation. **(c)** The comparison of coagulative necrotic volume of tumor tissues in each group after HIFU irradiation. (**d**) The comparison of EEF values of tumor tissues in each group after HIFU irradiation. **(e)** HE staining of tumor tissues of each group after HIFU irradiation (100 × magnification). **(f)** TTC staining of each group of tumor tissues after HIFU irradiation, Greyish white areas represent coagulative necrosis caused by HIFU irradiation.

HIFU was used to ablate the tumor tissue under specific ablation parameters (sound power 120 W, exposure time 3 s). Immediately after HIFU irradiation, varying degrees of grayscale and echo intensity changes were clearly observed within the focal volume (Fig. 4a). The changes in the grey-scale value from before to after irradiation obtained for the *B. longum* group were greater than that found for the PBS group at the same time points (*P* = 0.02; *P* = 0.003; Fig. 4b). When the HIFU ablation volume was compared, we observed that the boundary between the irradiated and unirradiated areas was clear (Fig. 4e) and the *B. longum* group exhibited a greater volume of coagulative necrosis(P = 0.004; P = 0.01; Fig. 4c) and a lower energy efficiency factor (EEF) (P = 0.04; P = 0.015; Fig. 4d) compared with the PBS group. Compared with the two *B. longum* group at 1 and 2 weeks, under the same conditions, after HIFU irradiation, the *B. longum* group 7 days after the first injection had the larger coagulative necrosis volume (P = 0.027; Fig. 4c) and lower EEF value (P = 0.032; Fig. 4d), showing the stronger synergistic effect. In summary, the HIFU ablation effect of *B. longum* group was greater than that of PBS group, and the ablation efficiency of HIFU was the strongest 7 days after the injection of Bifidobacteria, which indicated that the effect of Bifdobacterium on the AET can synergistic HIFU ablation and the injection time has also an effect on the synergy. HE staining showed varying degrees of damage within the ablation area. The HE staining analysis of the *B. longum* group revealed that the distinction between the ablation and non-ablation zones was obvious. Severe damage to the ablation zone (i.e., nuclear pyknosis, fragmentation, and lysis) was observed, and the red staining was uniformly distributed. In the PBS group, the ablation and boundary zones showed scattering cell fragments, which indicated that this group exhibited a low level and less severe tissue damage compared with the *B. longum* group (Fig. 4f). The images further demonstrated that the Bifdobacterium alter the AET, resulting in more efficient HIFU ablation.

## Discussion

Seven days after the first injection, some blue-violet-stained Bifidobacteria were found scattered in the liver in addition to the tumor. This finding indicates the possibility that Bifidobacteria were retained by the intracellular reticuloendothelial system of the liver, increasing the residence time of Bifidobacteria in the liver. Over time, *B. longum* disappeared from normal tissues, and 2 weeks after the first injection, these bacteria homed to and proliferated only within the tumor, which is consistent with the results reported by Cronin^19,32^. Yazawa^20^ also conducted a *B. longum* colonization experiment in mice with stronger immunity, and the experimental results were consistent with the results of this paper. The mechanism through which Bifidobacteria specifically grow in tumors has not yet been elucidated. Yong^33^ proposed that Bifidobacteria enter tumors due to the rich nutrition, the complex and diverse immune microenvironment, and the local microvascular destruction found in tumor tissues, which are features that provide shelters that allow a small number of bacteria to escape the immune system^34,35^. Cronin^32^ confirmed that the number of bacteria in a tumor peaked on day 19. To avoid substantial errors in the measurement of tumors with large areas of necrosis caused by excessive tumor growth, all the samples included in this study were collected 1 or 2 weeks after the first injection. According to the results of this experiment, We found that 7 days after the frist injection, the effect of HIFU ablation was more effective. And related studies have confirmed that the bifidobacteria increases with time in the short term after injection^19^. So we gusse that the synergistic effect of HIFU is not directly related to the number of bifidobacteria transmitted, which may be related to the microenvironment changes (Changes of neovascularization and collagen fibers, etc.) we discuss below.

The AET is a factor that affects both ultrasound propagation and energy deposition, From the perspective of acoustic properties, changes in AET mainly alter the velocity and attenuation of sound in the tissue^36,37^, and these two factors are closely related to the tissue microenvironment. Therefore, this study examined differences in the velocity and attenuation of sound in tissue over various times after treatment with Bifidobacteria and PBS the perspective of tissue microenvironment. The results showed that the injection of Bifidobacteria increased the collagen fibre number, elastic modulus and sound velocity in tumor tissues, which is consistent with the proposed view that the ultrasonic propagation velocity, elastic modulus and collagen fibre content are closely related^36,37^ and confirms the effects of Bifidobacteria on the AET. However, the synthesis of collagen fibres is complicated, and thus, the mechanism through which Bifidobacteria regulate the changes in collagen fibres in tumor tissues has not yet been elucidated. Over time, the collagen fibre number showed significant decreases, and the elastic modulus and sound velocity did not show significant changes. Therefore, the elastic modulus and sound velocity might be related to not only the content of collagen fibres but also their arrangement^38^. However, in the *B. longum* group, no significant change in sound attenuation was detected after the injection of Bifidobacteria. The reasons are as follows: in this study, the attenuation we measured included the attenuation of absorption and scattering^36–38^. In addition to the absorption attenuation and scattering attenuation of collagen fibres, the new blood vessels still produced scattering attenuation. From the results, it can be seen that the neovascularization of the *B. longum* group is significantly less than that of the PBS group, so the scattering attenuation caused by the neovascularization is increased in the PBS group, and we thus hypothesized that the blood vessels in the PBS group induce increases in the attenuation of scattering, resulting in the total compensation of ultrasonic attenuation. However, whether the changes of tissue in the tumor can really cause ultrasonic energy deposition and enhance HIFU ablation. We further carried out the experiment of HIFU ablation.

Under the same HIFU irradiation intensity and exposure time, the changes in the grey-scale value and volume of coagulative necrosis found for the *B. longum* group were greater than that found for the PBS group, and the *B. longum* group exhibited a decreased EEF and a higher ablation efficiency compared with the PBS group. Therefore, treatment with Bifidobacteria increases the utilization of ultrasonic energy, and thus, the objective of synergistic HIFU treatment can be achieved. There are two reasons for this discovery. First of all, the increased ultrasonic energy utilization might be due to the fewer number of new blood vessels in the tissue, which is associated with a reduced amount of energy carried by the blood flow, and this effect is conducive to the deposition of sound energy. Secondly, The high content of collagen fibres is associated with higher tumor tissue hardness and an increased acoustic impedance difference, and these effects may increase thermal diffusion and thus the ability of tissue to convert ultrasonic energy absorption^35,39^. Thirdly, the increased deposition of Bifidobacteria may reduce the cavitation threshold of tumor tissues, increasing the ultrasound energy deposition, and then therapeutic effect of HIFU was increased^40^. In this manuscript, we collectively refer to the resulting HIFU ablation effect as HIFU ablation. In the manuscript, it is point irradiation and each tumor is irradiated only once. Therefore, we think that it is mainly the thermal effect, but there is the possibility of cavitation, for example, the presence of Bifidobacterium may reduce the cavitation threshold of the tumor and increase the deposition of sound energy. In future work, the temperature measurements and cavitation cavitation of HIFU targeting regions will be increased to further confirm this conjecture. Seven days after the injection of Bifidobacteria, i.e., 7 days of bacterial action on tumor tissue, an improved effect on ablation was obtained, which was consistent with the change in the collagen fibre content. We think that collagen fibers may play an important role in HIFU ablation. However, there were many influencing factors, and further studies should thus explore the specific reasons.

This study confirmed that Bifidobacteria targeted tumor tissue, reduced tumor angiogenesis, and promoted the synthesis of collagen fibres, and these effects induced changes in the hardness and acoustic properties of the tissue. The alterations in these tissue properties may led to an increased volume of necrosis in mouse tumors following HIFU, and we propose that the mechanism of this necrosis enhancement is essentially thermal, and tumor tissue necrosis may lead to the change of cavitation threshold, that is, there is a certain cavitation effect. Therefore, the efficiency of HIFU treatment is improved from these two aspects.In addition, prolonging the time that Bifidobacteria can act on tissue did not improve the results of HIFU therapy. After injection of Bifidobacteria, the increase of collagen fibres may increase the production of more aggressive tumor phenotype and increase the difficulty of clinical treatment. However, it is well known that tumor growth and metastasis are mainly dependent on angiogenesis. The new type of intratumoral blood vessels provide nutrition and oxygen for tumor cells, which is the main way for tumor cells to enter the circulation and metastasize to distant organs. In the manuscript, although the collagen fibres in the tumor increased, new angiogenesis decreased. Therefore, whether Bifidobacteria injection within 7 days will lead to more aggressive tumor phenotype, we will further verify. And in follow-up experiments, we will also optimize the Bifidobacteria injection dose and bifidobacteriua residence time in the tumor. Based on the results obtained in this study, the proposed synergistic approach using Bifidobacteria as a biotargeting material represents a new, safe, and non-invasive foundation for the development of novel therapeutic synergists.

## Methods

### Ethics statement

All animal experimental protocols were reviewed and approved by the Chongqing Medical University Animal Care Committee. The methods used for the experiments were in accordance with the approved guidelines and the ethical standards stated in the Declaration of Helsinki.

### Strains, experimental animals and main materials

*B. longum* ATCC-15707 was provided by the Basic Medical College of Chongqing Medical University. Female BALB/c nude mice (strain nu/nu, aged 4 weeks, and weighing 15–20 g) were purchased from Beijing Hua Fukang Biotechnology Co., Ltd.. MDA-MB-231 human breast cancer cells were provided by the Institute of Ultrasound Imaging, Chongqing Medical University. A high-energy ultrasonic testing system (RAM-500 SNAP, RITEC) was purchased from Beijing Ritai Technology Co., Ltd., and this system included a digital oscilloscope, two immersion probes, and two immersion probe lines. The tissue hardness was tested using an Instron material testing machine (E1000, College of Bioengineering, Chongqing University). The therapeutic transducer of the JC-200 focused ultrasound tumor treatment system (Chongqing Haifu Medical Technology Co., Ltd., Chongqing, China) had a focal length of 145 mm, a diameter of 220 mm, and a working frequency of 0.94.

### B. longum culture

*B. longum* isolated from the human body was used in this study. Frozen *B. longum* was activated and added to a 15-ml sterile centrifuge tube containing 10 ml of MRS medium. The 15-ml centrifuge tube was placed in a sealed bag containing a gas-producing anaerobic bag for anaerobic culture at 37°C for 12 h. The Bifdobacterium was harvested by centrifuging at 1000 rpm for 10 min at 4 °C. The Bifdobacterium was diluted with phosphate buffered saline (PBS) (pH 7.4), and the concentration of Bifdobacterium was adjusted to about 4 × 10^8^ bacilli/ml

### Establishment of tumor-bearing model mice

MDA-MB-231 cells (human breast cancer cells) were cultured, and during the logarithmic phase of growth, the cells were digested with trypsin and centrifuged at 1000 rpm for 5 min. The cell concentration was adjusted to 5 × 10^6^ cells/ml in PBS. BALB/c nude mice were injected with 200 µl of the above-mentioned cell suspension into the right thigh. The tumor formation rate was 95%. After approximately 20 days, tumors with a diameter of approximately 0.8–1 cm were used for subsequent studies.

### Grouping and methods

All tumor-bearing mice were randomly divided into two groups: the *B. longum* group (n = 40) and the PBS group (n = 40). The mice belonging to the *B. longum* group were injected with the *B. longum* suspension for 3 consecutive days via the tail vein (200 µl per day at a concentration of 4 × 10^8^ bacilli/ml). The mice belonging to the PBS group were injected with PBS for 3 consecutive days (200 µl per day). Twenty mice from each group were used for measurements of the AET and observations of the targeting of *B. longum* to tumor tissue at weeks 1 (n = 10) and 2 (n = 10) after the first injection, and 20 mice from each group were subjected to HIFU treatment at weeks 1 (n = 10) and 2 (n = 10) after the first injection.

### Specific targeting of *B. longum* to tumor

The nude mice used for testing the acoustic environment 1 and 2 weeks after the first injection were also used for harvesting the heart, liver, spleen, lungs, kidneys and part of the tumor. The collected samples were fixed in 4% paraformaldehyde and subjected to Gram staining.

### Effect of *B. longum* on the acoustic environment in tumors

**Figure 5:**
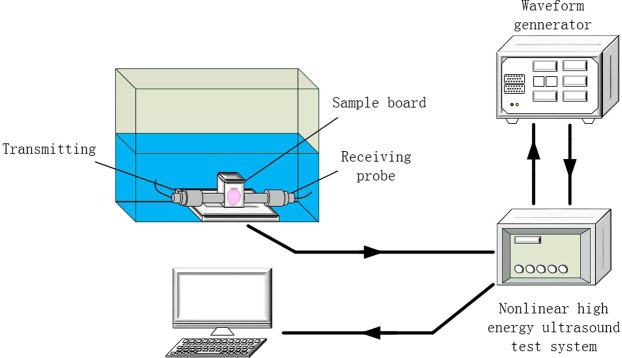
Schematic illustration of the devices for detection of the acoustic properties (sound velocity and sound attenuation) of tumor tissue.

Subcutaneous tumor extraction and tumor tissue acoustic property detection (sound velocity and attenuation): Ten tumor-bearing mice in each group were sacrificed 1 and 2 weeks after the injection. After the induction of anaesthesia, the nude mice were sacrificed, and the tumors were completely excised. A portion of the tumor tissue was fixed in 4% paraformaldehyde for sectioning, and the remaining portion was immediately placed in physiological saline at 4°C for subsequent use. The acoustic properties of the tumor tissue were measured using a plug-in pulse substitution method. Two 1-MHz immersion probes, which faced each other in a coaxial direction and were fixed on either side of the custom tool, were used. The sample was placed in the specimen plate of the system and immersed in degassed water, and the oscilloscope and nonlinear high-energy ultrasonic test system were adjusted (Fig. 5). The transit time (Δ_transit time_) of the ultrasound in the sample, the amplitude of the first echo of the acoustic signal, and the amplitude at time zero were measured three times, and the sound velocity and attenuation coefficient of the tumor tissue sample were then calculated according to the following formulas:

1 $${\rm{S}}{\rm{o}}{\rm{u}}{\rm{n}}{\rm{d}}\,{\rm{v}}{\rm{e}}{\rm{l}}{\rm{o}}{\rm{c}}{\rm{i}}{\rm{t}}{\rm{y}}:C=\frac{l}{\Delta {\rm{t}}{\rm{r}}{\rm{a}}{\rm{n}}{\rm{s}}{\rm{i}}{\rm{t}}\,{\rm{t}}{\rm{i}}{\rm{m}}{\rm{e}}}{\rm{m}}/{\rm{s}}$$

where $l$ is the average from three thickness measurements of the sample and Δ_transit time_ is the average from three measured transit times obtained for the sample.

2 $${\rm{Sound}}\,{\rm{attenuation}}:{Attenuation}=\frac{1}{l}log\left(\frac{{A}_{2}}{{A}_{1}}\right)\,{\rm{dB}}/{\rm{cm}}$$

where A_1_ and A_2_ are the response amplitudes at time zero and of the first echo, respectively.

Biomechanical testing: The tumor samples used for the detection of sound velocity and attenuation were immediately placed in an ice bath at 4 °C and sent to the Bioengineering Institute of Chongqing University for biomechanical analysis using an Instron material testing machine. The tumor tissue temperature was re-heated to physiological temperature, 37 °C, and placed on the operating table. Compressive force was loaded along the axial direction of the cylinder at a loading rate of 1 mm/min, and each specimen was measured only once. Force (N)-displacement ($\Delta l$) datasets were obtained for each tumor sample, and the force and displacement data for each specimen were used to calculate the stress and strain of the tumor tissue using the following equations: $Stress=\frac{N}{S}\,$and $Strain=\frac{\Delta l}{l}$, where *S* is the surface area of the tumor in contact with the compression cylinder, $S=\pi {\left(\frac{d}{2}\right)}^{2}$, and *d* is tumor diameter. The corresponding stress-strain curve was created using Origin 8.0 software. The x-axis represents the strain, and the y-axis represents the stress. After identifying the linear part of the stress-strain curve, its primary coefficient, which is the elastic modulus (E) of the tumor tissue, was determined.

Histopathological detection: All tumor samples were subjected to pathology and immunohistochemistry evaluations. Masson staining was used to observe the collagen fibres in the tumor tissues. Four regions were randomly selected and imaged using an Olympus BX51 microscope at 200× magnification. The collagen fibres were stained blue. The acquired images were analysed using Image Pro Plus 6.0, and the average percentage of collagen fibres in the four regions was calculated as the percentage of the entire image. Cluster of differentiation 31 (CD_31_) is a marker of vascular endothelial differentiation and can be used for the assessment of neovascularization in tumors.

### HIFU ablation

7 and 14 days after the first injection, HIFU ablation was performed on 10 tumor-bearing mice belonging to each group, respectively. Nude mice were anaesthetized, the tumor site was completely immersed in degassed water, and the treatment area was selected under ultrasound guidance. HIFU ablation was performed through single point irradiation with an output power of 120 W for 3 s. The average grey-scale values in the ablation zone before and after ablation were recorded and compared using Grey Val 1.0 software (Chongqing Haifu Medical Technology Co., Ltd., Chongqing, China). Twenty-four hours after HIFU irradiation, the tumor-bearing mice were sacrificed. The tumor was removed by dissecting along the direction of HIFU irradiation. Half of the tumor tissue was placed in 4% paraformaldehyde for HE staining to observe the degree of tumor necrosis. The remaining half was placed in a TTC stain at 37 °C, and the coagulative necrotic volume (V) was measured using irregular volume measurement software. The formula $EEF=\frac{{\rm{\eta }}{\rm{Pt}}}{{\rm{V}}}\,$(*η* is the focusing coefficient of HIFU, P is the acoustic power, and t is the irradiation duration time) was used to calculate the EEF, which represents the ultrasonic energy required to damage a unit volume of tumor. Therefore, a smaller EEF is associated with a higher ultrasonic energy utilization rate.

### Statistical analysis

SPSS (IBM) was used for all data analyses. The measurement data are expressed as the mean ± standard deviation (SD). The normality of the data was tested using the Shapiro–Wilk test, and the Student–Newman–Keuls (SNK) method was used for comparisons between two groups. P<0.05 was considered statistically significant in all cases.

## Data Availability

The datasets generated and analysed during the current study are available from the corresponding author.
